# Prognostic significance of LINE-1 hypomethylation in oropharyngeal squamous cell carcinoma

**DOI:** 10.1186/s13148-017-0357-z

**Published:** 2017-05-30

**Authors:** Carlo Furlan, Jerry Polesel, Luigi Barzan, Giovanni Franchin, Sandro Sulfaro, Salvatore Romeo, Francesca Colizzi, Aurora Rizzo, Vittorio Baggio, Vittorio Giacomarra, Angelo Paolo Dei Tos, Paolo Boscolo-Rizzo, Emanuela Vaccher, Riccardo Dolcetti, Luca Sigalotti, Elisabetta Fratta

**Affiliations:** 1 Division of Radiotherapy, Centro di Riferimento Oncologico, IRCCS-National Cancer Institute, Aviano, PN Italy; 2 Unit of Cancer Epidemiology, Centro di Riferimento Oncologico, IRCCS-National Cancer Institute, Aviano, PN Italy; 3 Department of Surgery, Centro di Riferimento Oncologico, IRCCS-National Cancer Institute, Aviano, PN Italy; 4 Division of Pathology, General Hospital “S. Maria degli Angeli”, Pordenone, Italy; 5 Department of Pathology, San Donà di Piave Hospital, San Donà di Piave, Italy; 6 Immunopathology and Cancer Biomarkers, Centro di Riferimento Oncologico, IRCCS-National Cancer Institute, Aviano, PN Italy; 7 Department of Radiation Oncology, Treviso Regional Hospital, Treviso, Italy; 8 Unit of Otolaryngology, General Hospital “S. Maria degli Angeli”, Pordenone, Italy; 9 Department of Diagnostic Pathology, Treviso Regional Hospital, Treviso, Italy; 10 Department of Neurosciences, ENT Clinic and Regional Center for Head and Neck Cancer, Treviso Regional Hospital, University of Padua, Treviso, Italy; 11 Division of Medical Oncology A, Centro di Riferimento Oncologico, IRCCS-National Cancer Institute, Aviano, PN Italy; 12 Translational Research Institute, The University of Queensland Diamantina Institute, Brisbane, Australia; 13 Institute of Clinical Pathology, University Hospital of Udine, Udine, Italy

**Keywords:** Oropharyngeal squamous cell carcinoma, DNA methylation, LINE-1, HPV

## Abstract

**Background:**

Inclusion of new biomarkers to improve a personalized treatment approach for oropharyngeal squamous cell carcinoma (OPSCC) is urgently needed. Hypomethylation of the Long interspersed nucleotide element-1 (LINE-1) repetitive elements, a widely accepted surrogate of overall genomic DNA methylation content, was found to be associated with a poor prognosis in several cancers. At present, no studies have investigated the influence of LINE-1 methylation levels on OPSCC relapse. The main goal of this study was the evaluation of the prognostic value of LINE-1 methylation status in predicting early tumor relapse in locally advanced OPSCC.

**Methods:**

We retrospectively reviewed a cohort of 77 patients with stage III–IVB OPSCC. Methylation of LINE-1 repetitive sequences was evaluated by real-time quantitative methylation-specific PCR in formalin-fixed paraffin-embedded tissues. The prognostic relevance of LINE-1 methylation was assessed by comparing patients who relapsed within 2 years from the end of treatment (cases) with those who did not (controls). Results were validated in an independent cohort of 33 patients with OPSCC.

**Results:**

With respect to early OPSCC relapse, the mean LINE-1 methylation level was significantly lower in relapsed cases than in control group (*p* < 0.01). Interestingly, LINE-1 methylation was lower in relapsed cases than in controls in both HPV16-negative and HPV16-positive OPSCC patients, even if statistical significance was reached only for the former group (*p* = 0.01). LINE-1 methylation levels were also significantly reduced in relapsed cases with respect to the controls in OPSCC current smokers (*p* = 0.02). Consistently, in HPV16-negative current smokers, OPSCC relapse was significantly associated with decreased levels of LINE-1 methylation (*p* = 0.02). Using logistic regression model, we found that patients with hypomethylated LINE-1 were associated with a 3.5 higher risk of early relapse than hypermethylated ones (OR = 3.51; 95% CI 1.03–12.00). Adjustment for potential confounders did not substantially change the risk magnitude. Results from the validation cohort confirmed the lower LINE-1 methylation in patients who early relapsed compared to relapse-free patients.

**Conclusions:**

LINE-1 hypomethylation is associated with higher risk of early relapse in stage III–IVB OPSCC. Further validation in a prospective study is needed for its application in daily clinical practice.

## Background

Head and neck cancers rank as the sixth most frequent cancer worldwide with the most common type being the head and neck squamous cell carcinoma (HNSCC). Up to 30% of HNSCC is represented by oropharyngeal squamous cell carcinoma (OPSCC), which originates in the tonsils, base of the tongue, soft palate, and posterior pharyngeal wall, and accounts for approximately 123,000 incident cases diagnosed worldwide each year [1]. Tobacco use and heavy alcohol consumption represent the main risk factors for HNSCC development, and they can act synergistically to increase the risk of this malignancy [2]. In the last years, the incidence of HNSCC has been decreasing in developed countries due to a reduction in tobacco exposure. Nevertheless, there is a concomitant increase in the incidence of OPSCC as a result of transforming infections by high-risk alpha human papillomavirus (HR α-HPV) types [3, 4]. HR α-HPVs are sexually transmitted viruses which can affect the crypt epithelium of the palatine and lingual tonsils, ultimately leading to a malignant phenotype. HPV-driven OPSCC predominantly affects young men and typically non-smokers. High-risk HPV is present in 45–90% of OPSCC, but this figure has been reported to dramatically vary according to geographic area [3, 5].

OPSCC is frequently diagnosed when already symptomatic and, therefore, in advanced stages. The treatment of OPSCC frequently requires a multimodality approach that may include surgery, radiotherapy (RT), and chemotherapy administered in different combinations [4]. Treatment decision for OPSCC patients requires consideration of tumor subsite and stage, functional outcomes and morbidity associated with various treatment approaches, and patient-specific factors such as performance status, comorbidities, and preference. However, as different combined treatments can affect the quality of life, the need to identify biomarkers that could guide treatment decisions and/or predict relapse of OPSCC patients is a pressing issue. In particular, relapse develops in a considerable number of locally advanced OPSCC patients, and the risk of locoregional relapse after radical treatment varies between 20 and 52% [6]. Despite recent improvements in both surgical and radiotherapy treatments, OPSCC relapse is for the most part a time-limited phenomenon, since the majority of recurrences appears within the first 2 years after treatment for both HPV-positive (66.0%) and HPV-negative (89.3%) OPSCC patients [7]. Additionally, OPSCC patients who relapse within 1 year of initial treatment have a significantly worse prognosis and are generally thought to have radiation-resistant tumors [8]. Interestingly, OPCSS relapse usually occurs earlier in HPV-negative than in HPV-positive patients (median time to recurrence 9.9 vs. 19.6 months) [7]. Along this line, it has been reported that patients with HPV-positive OPSCC have more favorable outcomes than those with HPV-negative OPSCC [9–12] probably due to a stimulation of the immune response directed to HPV-specific antigens that may play a role in the improved response to therapy [13–15]. Although HPV positivity represents a strong prognostic factor for both improved survival and reduced risk of relapse, there is a subset of HPV-positive OPSCC patients who still experience poor outcomes [16]. Furthermore, HPV-negative OPSCC patients, who have an even higher risk of relapse despite intensive therapy, are still lacking suitable prognostic biomarkers for clinical outcome prediction. From the above considerations, it emerges that the identification of new prognostic molecular markers is urgently needed to more precisely identify OPSCC patients at higher risk of early relapse.

The biological diversity that characterizes tumor subsets with different prognosis is an important background for the identification of molecular markers of potential clinical utility. In this regard, several studies focused on mechanisms underlying the differences in clinical and molecular behavior between HPV-positive and HPV-negative OPSCC [16–18]. Recent data suggest that epigenetic changes deeply influence the biology of OPSCC and may contribute, at least in part, to the different therapeutic responses shown by HPV-positive and HPV-negative OPSCC [19]. In this context, methylation of genomic DNA at CpG dinucleotides undoubtedly represents one of the most extensively characterized epigenetic regulators of gene expression [20]. Aberrant DNA hypermethylation represents an early and frequent molecular event during multistep carcinogenesis in OPSCC, especially in long-term tobacco users, along with prolonged alcohol consumption [21–23]. However, a high frequency of promoter DNA hypermethylation has also been observed in smokeless tobacco-associated OPSCC tissue and in corresponding adjacent normal mucosa [24]. Besides gene-specific hypermethylation, genome-wide hypomethylation might contribute to tumorigenesis and cancer progression by promoting genomic instability, reactivating endogenous parasitic sequences, and inducing the expression of oncogenes [25]. Extensive DNA hypomethylation in tumors occurs frequently at repetitive sequences, including short and long interspersed nuclear elements, segmental duplications, and sub-telomeric regions [26]. These findings further validate the role of DNA methylation in maintaining the stability of the human genome and the suppression of transposable elements in mammalian cells.

The increasing role of aberrant methylation in OPSCC biology strongly suggests for the opportunity to test methylation markers as potential indicators of disease prognosis. In addition to studies investigating the prognostic role of the methylation status of specific genes, the study of the methylome, which encompasses the totality of the genomic DNA methylated sites, is gaining ever growing interest due to its promising value as prognostic marker in human cancer [27, 28]. Along this line, hypomethylation of the long interspersed nucleotide element-1 (LINE-1) repetitive elements, a widely accepted surrogate of overall genomic DNA methylation content, has been associated with a poorer overall and/or progression-free survival in many different tumor types [29–34]. However, LINE-1 hypomethylation also correlated with improved overall survival in several malignancies, thus suggesting that the underlying biological effects of LINE-1 hypomethylation on patient outcome may depend on tumor histotype [35, 36]. In addition to its potential role as a prognostic biomarker, LINE-1 methylation level may be useful for assessing the risk of cancer relapse and/or predicting the therapeutic efficacy of cancer treatment regimens [37]. Although several studies have evaluated the prognostic role of LINE-1 methylation in HNSCC, their results are still inconclusive [38–41]. Furthermore, no prior study has specifically investigated the influence of the overall level of genomic DNA methylation on early OPSCC relapse risk. On these grounds, we designed a retrospective case-control study aimed at evaluating whether LINE-1 methylation could represent a useful prognostic molecular marker in predicting early tumor relapse in locally advanced OPSCC, in order to improve the clinical management of the disease.

## Methods

### Patients

We retrospectively reviewed the records of 104 patients who fulfilled the following inclusion criteria: (i) stage III–IVB OPSCC; (ii) managed with curative intent with radiotherapy ± chemotherapy or radiotherapy + surgery ± chemotherapy, in accordance with current clinical practice; (iii) treated between 2001 and 2013 at the National Cancer Institute of Aviano (Italy). Clinical data, socio-demographic characteristics, smoking habits, and treatment were retrieved from medical records. However, 27 patients were excluded because of inadequate tumor tissue sample or incomplete clinical data, thus leaving 77 patients eligible for the final analysis. The treatment planning with respect to surgical vs. nonsurgical approaches was discussed by a multidisciplinary tumor board for optimal decision-making. Treatment policies were based on offering definitive chemo-radiotherapy to patients with stage III–IVB OPSCC who were not suitable for conservative surgery. Patients were followed at regular intervals to determine tumor status until death or 31 December 2015, whichever came first. Early relapse was defined as that diagnosed in the first 2 years after curative intent. Twenty-eight patients who relapsed within 24 months from the end of the treatment (cases) were compared to those who were relapse-free for at least 24 months after the treatment (controls, *n* = 49). A separate validation cohort of 33 patients who underwent treatment from 2003 to 2014 was then recruited from the Treviso Regional Hospital. Treatment planning, treatment delivery, and follow-up strategies were applied in the same manner as in the discovery cohort. All tumors were reclassified according to the American Joint Committee on Cancer 7th Edition [42].

In both discovery and validation cohorts, a representative neoplastic sample was collected for each patient at the time of surgical resection or biopsy from a non-necrotic area of the carcinoma. Samples from OPSCC patients were taken before starting any treatments. Hematoxylin- and eosin-stained slides of the tumors were reviewed by the pathologist, who marked the areas of the tumor and adjacent non-neoplastic tissue. The study was limited to neoplastic lesion that contained ≥70% neoplastic cells. The study was approved by the local Independent Ethic Committees. Participants provided written informed consent for inclusion in the study.

### Quantitation of HPV16 E6 DNA using real-time quantitative PCR analysis

Genomic DNA was extracted from OPSCC formalin-fixed paraffin-embedded (FFPE) tissues using the QIAamp DNA FFPE Tissue Kit (Qiagen), following the manufacturer’s protocol. DNA concentration was measured with the Qubit Fluorometer using the Qubit dsDNA High Sensitivity Assay Kit (Life Technologies). The quantification of HPV16 DNA was performed with the ABI prism 7700 Sequence Detection System (Life Technologies) by using specific primers for the amplification of a region spanning the E6 gene of the HPV16 genome [40]. SYBR green quantitative HPV16-PCR reactions were performed in triplicate on 10 ng of DNA in a final volume of 25 μl 1 X SYBR Green Master Mix (Life Technologies) at 95 °C for 10 min, followed by 45 cycles at 95 °C for 15 s and at 60 °C for 1 min, and dissociation performed at 95 °C for 15 s, 60 °C for 20 s, and 95 °C for 15 s. SYBR Green primer sets were as follows: HPV16 E6, forward CTGCAATGTTTCAGGACCCA and reverse TCATGTATAGTTGTTTGCAGCTCTG. Known amounts of E6 DNA molecules were used to generate an absolute standard curve. The copy number of E6 DNA was determined in each sample by extrapolation of the standard curve. E6 copy number ≥100 copy/well was regarded as positive.

### Quantitative methylation-specific PCR analysis for the methylation levels of LINE-1

Genomic DNA was obtained from OPSCC FFPE tissues in quantities sufficient for bisulfite treatment. Bisulfite conversion was carried out on 500 ng genomic DNA using EZ DNA Methylation-Gold™ Kit (Zymo Research), according to the manufacturer’s protocol. SYBR Green quantitative methylation-specific PCR (qMSP) was performed with the ABI prism 7700 Sequence Detection System (Life Technologies). QMSP reactions were carried out in triplicate on 2 μl of bisulfite-modified genomic DNA in a final volume of 25 μl 1 X SYBR Green Master Mix (Life Technologies), as described above. LINE-1 qMSP reactions were run in parallel with SYBR Green primers for methylated (forward, 5′-CGCGAGTCGAAGTAGGGC-3′; reverse, 5′-ACCCGATTTTCCAAATACGACCG-3′) and for unmethylated sequence (forward, 5′-TGTGTGTGAGTTGAAGTAGGGT-3′; reverse, 5′-ACCCAATTTTCCAAATACAACCATCA-3′) [43]. Known amounts of LINE-1 methylated and unmethylated DNA molecules were used to generate absolute standard curves. The copy number of methylated or unmethylated sequences for each target gene was established by extrapolation from the standard curves. The percentage of methylation was defined as the ratio between methylated molecules and the sum of methylated and unmethylated molecules.

### Statistical analysis

Despite recurrence is a time-dependent outcome, in head and neck cancer patients, early recurrence (i.e., within 2 years from end of treatment) is a major predictor of poor prognosis, with little impact of the time this event occurs [7, 8]. Therefore, in the present case-control study, we focused on the early recurrence itself rather than on the time to relapse, considering it as a binary variable. As a consequence, patients were followed up for a maximum of 2 years after the end of treatment.

Cases and controls were compared in terms of socio-demographic characteristics (age and gender), lifestyle (smoking status), clinical (tumor size, number of lymph nodes involved, and clinical stage), and pathologic factors (HPV16 status). All variables were categorized, and cases and controls were compared using Fishers’ exact test. LINE-1 methylation levels between cases and controls and across strata were compared using the non-parametric Kruskal–Wallis test. The prognostic relevance of LINE-1 methylation and other covariates on OPSCC relapse was assessed by calculation of odds ratios (OR) and corresponding 95% confidence intervals (CI) through univariate and multivariate logistic regression model. The statistical analyses were carried out using *R* Statistical Software (Foundation for Statistical Computing, Vienna, Austria) for Windows. All statistical tests were two-sided, and significance was claimed for *p* ≤ 0.05.

The association between LINE-1 methylation and cancer relapse was further investigated across cancer risk categories. Using information on HPV16 status and smoking habits (current smoking was considered as a proxy of smoking >20 pack year), OPSCC were categorized as low risk of early relapse (non-smokers HPV16-positive), intermediate risk (smokers HPV16-positive or non-smokers HPV16-negative), and high risk (smokers HPV16-negative).

## Results

### Patients

The distributions of socio-demographic and clinico-pathologic characteristics among cases and controls at baseline are presented in Table 1. In the discovery set, the median age of OPSCC patients was 59 (range 41–87 years) and the majority of cases (67.9%) and controls (67.4%) were men with a similar gender distribution between the two groups. Within the discovery cohort, 49 patients (64%) were classified as current smokers, while 14 (18%) were former smokers, and 14 (18%) have never smoked. The HPV16 E6 DNA region was detected in 18 of 77 OPSCC patients (23.4%). Although HPV16 infection appeared to have no effect on the likelihood of early tumor relapse (*p* = 0.58), the percentage of OPSCC patients positive for HPV16 E6 DNA was higher in controls when compared to cases. There was no statistically significant difference between the two groups regarding treatment approaches, and also, all the other socio-demographic and clinico-pathologic features included in the analysis showed no statistically significant differences (Table 1).

**Table 1:** Distribution of socio-demographic and clinical features, odds ratio (OR) of relapse, and corresponding 95% confidence intervals (CI) in 28 OPSCC patients who recurred within 24 months from end of treatment (cases) and 49 OPSCC patients who did not (controls)

Variables	Cases, *n* = 28		Controls, *n* = 49		OR (95% CI)^a^	OR (95% CI)^b^
	*n*	(%)	*n*	(%)		
Gender						
Male	19	(67.9)	33	(67.4)	1^f^	1^f^
Female	9	(32.1)	16	(32.7)	1.07 (0.38–2.97)	1.08 (0.37–3.18)
Age (years)						
<55	10	(35.7)	18	(36.7)	1^f^	1^f^
55–59	10	(35.7)	12	(24.5)	1.52 (0.47–4.91)	1.66 (0.49–5.66)
≥60	8	(28.6)	19	(38.8)	0.76 (0.25–2.37)	0.90 (0.27–3.00)
Smoking habits						
Never	4	(14.3)	10	(20.4)	1^f^	1^f^
Former	3	(10.7)	11	(22.5)	0.62 (0.11–3.62)	0.66 (0.11–4.12)
Current	21	(75.0)	28	(57.1)	1.73 (0.46–6.54)	1.44 (0.32–6.45)
N^c, d^						
0–1	5	(17.9)	15	(31.3)	1^f^	1^f^
2–3	23	(82.1)	33	(68.7)	2.02 (0.63–6.55)	1.12 (0.42–2.99)
T^c, d^						
1–2	11	(39.3)	31	(64.6)	1^f^	1^f^
3–4	17	(60.7)	17	(35.4)	3.23 (1.18–8.86)	1.92 (1.02–3.62)
Stage						
III	4	(13.8)	12	(25.0)	1^f^	1^f^
IV	25	(86.2)	36	(75.0)	2.77 (0.68–11.30)	2.44 (0.58–10.29)
HPV16						
Negative	23	(82.1)	36	(73.5)	1^f^	1^e^
Positive	5	(17.9)	13	(26.5)	0.59 (0.18–1.93)	0.92 (0.24–3.61)
Therapy^c^						
RT ± CT	16	(57.1)	21	(42.9)	1^f^	1^f^
RT + CH ± CT	12	(42.9)	28	(57.1)	0.53 (0.20–1.45)	0.48 (0.16–1.51)
LINE-1 methylation						
≥70%	6	(21.4)	19	(38.8)	1^f^	1^f^
50 to <70%	9	(32.1)	17	(34.7)	1.68 (0.48–5.83)	2.18 (0.53–8.97)
<50%	13	(46.4)	13	(26.5)	3.51 (1.03–12.00)	4.12 (1.01–16.91)
Continuous 10% decrease^e^					1.45 (1.13–1.87)	1.50 (1.13–2.00)

^a^Estimated through logistic regression model adjusted for sex and age

^b^As footnote a, plus adjustment for smoking habits, stage, and HPV16

^c^ *N* number of lymph nodes involved, *T* tumor size, *RT* radiotherapy, *CT* chemotherapy, *CH* surgery

^d^The sum does not add up to total because of missing values

^e^LINE-1 methylation analyzed as continuous variable, risk associated with 10% decrease in methylation

^f^Reference category

### LINE-1 methylation in OPSCC patients

**Fig. 1:**
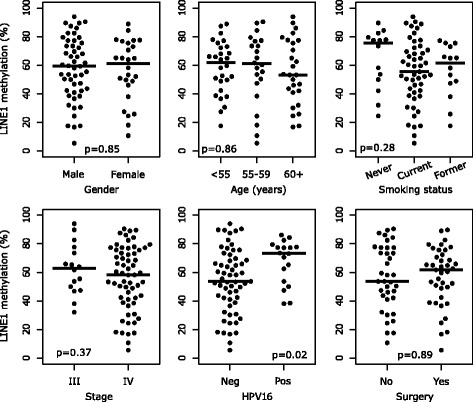
LINE-1 methylation according to socio-demographic characteristics, smoking habits, tumor features, and surgical treatment. *Black horizontal bars* represent the median values of LINE-1 methylation for each group. *p* values were determined by the non-parametric Kruskal–Wallis test

**Fig. 2:**
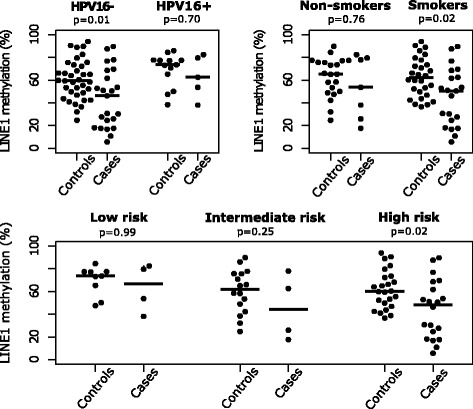
LINE-1 methylation in patients who relapsed within 24 months from the end of treatment (cases) and in patients who did not (controls), according to HPV16 status, current tobacco smoking, and risk group. Risk group was defined on HPV16 status and current tobacco smoking as follow: low (HPV16+/non-smokers), intermediate (HPV16−/non-smokers or HPV16+/smokers), high (HPV16−/smokers). *Black horizontal bars* represent the median values of LINE-1 methylation for each group. *p* values were determined by the non-parametric Kruskal–Wallis test

Methylation of LINE-1 repetitive elements was evaluated by qMSP analysis in DNA obtained from 77 OPSCC tissues. LINE-1 methylation levels were largely heterogeneous (range, 6–94%) and were not correlated with age, gender, tumor stage, and treatment administered (Fig. 1). However, LINE-1 methylation level was significantly higher in HPV16-positive OPSCC patients than in HPV16-negative ones (median, 67.5 and 53.9%, respectively; *p* = 0.02). Therefore, the interaction between LINE-1 methylation levels and HPV16 infection was further investigated according to their HPV status (Fig. 2). Interestingly, LINE-1 methylation was lower in cases than in controls in both HPV16-negative and HPV16-positive, even if statistical significance was reached only among the former group (*p* = 0.01). Similarly, LINE-1 methylation was lower in cases than controls in smokers (*p* = 0.02). Conversely, no statistically significant difference in LINE-1 methylation was found between cases and controls among non-smokers OPSCC patients (Fig. 2), even if median level was lower in cases than in controls. Finally, stratification through HPV16 and smoking status into OPSCC early relapse risk groups revealed lower levels of LINE-1 methylation in OPSCC cases belonging to the intermediate- and high-risk groups, even though the statistical significance was observed only in the high-risk group (*p* = 0.02) (Fig. 2).

### The impact of LINE-1 methylation on outcome

A logistic regression analysis was performed to investigate the association of LINE-1 methylation levels and 2-year OPSCC relapse. LINE-1 methylation level <50% was associated to a higher risk of early disease relapse (OR = 3.51; 95% CI 1.03–12.00) compared to ≥70%. All other variables evaluated did not significantly associate with outcome, though a trend towards an increased risk (*p* = 0.11) was observed for tumor stage. Adjustment for potential confounders did not substantially modify the risk magnitude, affecting only confidence intervals. LINE-1 hypomethylation was further analyzed as a continuous variable, reporting an increase in early relapse risk of 50% (95% CI 1.13–2.01) for every decrease of 10% in methylation (Table 1).

### Validation study

Study results were validated on an independent cohort of 33 OPSCC patients (Table 2). The validation set had similar distributions for most of the clinico-pathologic characteristics examined with respect to the discovery cohort. However, the validation cohort was older (*p* = 0.01), which included a higher proportion of T3–4 cancers (*p* < 0.01), and had a trend towards an increased prevalence of HPV16 infection (*p* = 0.11) than the discovery set.

**Table 2:** Distribution of socio-demographic and clinical features in 9 OPSCC patients who recurred within 24 months from end of treatment (cases) and 24 OPSCC patients who did not (controls) who served as validation set

Variable	Cases, *n* = 9		Controls, *n* = 24		*p* value^c^
	*n*	(%)	*n*	(%)	
Gender					
Male	4	(44.4)	17	(70.8)	0.23
Female	5	(55.6)	7	(29.2)	
Age, years					
<55	1	(11.1)	4	(16.7)	1.00
55–59	2	(22.2)	4	(16.7)	
≥60	6	(66.7)	16	(66.7)	
Smoking habits					
Never	3	(33.3)	5	(20.8)	0.32
Former	1	(11.1)	1	(4.2)	
Current	5	(55.6)	18	(75.0)	
N^a, b^					
0–1	3	(33.3)	9	(37.5)	1.00
2–3	6	(66.7)	15	(62.5)	
T^a, b^					
1–2	3	(33.3)	8	(33.3)	1.00
3–4	6	(66.7)	16	(66.7)	
Stage					
III	2	(22.2)	6	(25.0)	1.00
IV	7	(77.8)	18	(75.0)	
HPV16					
Negative	8	(88.9)	12	(50.0)	0.06
Positive	1	(11.1)	12	(50.0)	
Therapy					
RT ± CT	3	(33.3)	12	(50.0)	0.46
RT + CH ± CT	6	(66.7)	12	(50.0)	

^a^The sum does not add up to total because of missing values

^b^ *N* number of lymph nodes involved, *T* tumor size, *RT* radiotherapy, *CT* chemotherapy, *CH* surgery

^c^Fisher’s exact test

^d^Statistical comparison of the different characteristics in discovery and validation series

**Fig. 3:**
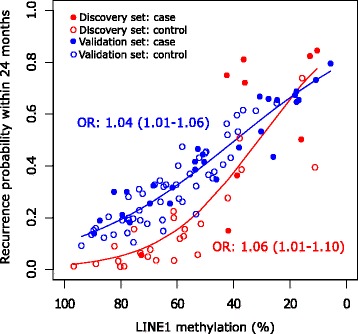
Predicted probability of relapse within 24 months from end of treatment in the discovery set and in the validation set

The probability of 2-year OPSCC relapse estimated according to the logistic model for case and controls is shown in Fig. 3 for both the discovery and the validation cohorts. In both cohorts, the risk of early relapse increases with decreasing LINE-1 methylation level. The estimated risks for each decrement of 1% were very similar, being 1.04 (95% CI 1.01–1.06) in the discovery set and 1.06 (95% CI 1.01–1.10) in the validation set.

## Discussion

This is the first study showing that methylation of LINE-1 repetitive elements predicts the risk of early post-treatment relapse in OPSCC patients. In fact, a progressively increasing risk of 2-year OPSCC relapse was significantly correlated with the decreasing of LINE-1 methylation. By including confounder adjustments, such as HPV16 infection and smoking status, we found that low LINE-1 methylation level maintained its ability to identify OPSCC patients at higher risk of early relapse. The positive validation of these results that we obtained on an independent cohort of OPSCC patients confirms the strength of LINE-1 hypomethylation as a reliable tool for identifying a subgroup of OPSCC patients with a high risk of early tumor relapse. As such, these results potentially bear an important clinical relevance both for improving treatment intensity decisions as well as for defining most appropriate follow-up procedures. Furthermore, the strength of the observed prognostic correlation is supported by the consideration that the study was carried out on a homogenous population comprising stage III–VIB OPSCC patients who have undergone radiotherapy ± chemotherapy or radiotherapy + surgery ± chemotherapy with curative intent.

**Fig. 4:**
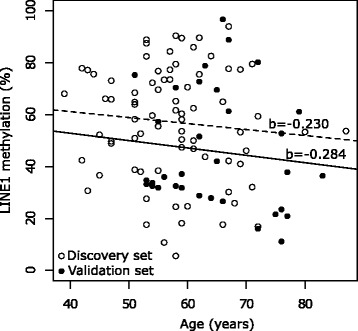
Relationships between LINE-1 methylation and age in the discovery and validation sets. The linear regression is represented by a *scattered line* (discovery set) and a *bold line* (validation set). Regression line coefficients (*b*) are also reported for both lines

Although there is growing evidence that global genomic DNA methylation decreases with aging [44–46], several studies did not observe an association between LINE-1 hypomethylation and age [47, 48]. Consistent with these data, we did not find any correlation between LINE-1 hypomethylation and age in both discovery and validation cohorts (Fig. 4). Similar to previous investigations [39–41], in our study, LINE-1 methylation status was different according to HPV16 infection. HPV-positive and HPV-negative OPSCCs are driven by different tumorigenic pathways [49], which are reflected in their diverse clinical behavior [16]. Several studies highlighted characteristic differences in gene expression profiles between HPV-negative and HPV-positive OPSCC, suggesting that a better clinical outcome may be partially due to HPV-related changes in the pattern of DNA methylation [19]. HPV-positive tumors showed increased methylation in the promoter region of tumor suppressor genes as compared to HPV-negative tumors, in which global genomic hypomethylation is more frequently observed [50]. Although the exact mechanism of global genomic DNA hypomethylation in HNSCC is still elusive, differences in the expression and/or activity of DNA methyltransferases (DNMTs) may explain HPV-related differences among OPSCC [41, 51]. In fact, evidence has been provided indicating that the E6 and E7 HPV16 oncoproteins can directly interact with DNMTs, thus stimulating their activity in vitro [52]. Furthermore, it has been reported that the HPV genome itself may become hypermethylated following infection or integration into host cells. This would represent a defense mechanism against foreign agents that may alter the expression patterns of HPV genes that are relevant for infection and transformation [53]. Accordingly, in our study, LINE-1 methylation status was different between HPV16-positive and HPV16-negative OPSCC patients, being lower in the latter. In this setting, the lowest level of LINE-1 element methylation was observed in HPV16-negative OPSCC patients who relapsed within 2 years (Fig. 2). Environmental factors, such as tobacco smoking, have been associated with global DNA hypomethylation. In fact, genotoxic exposure to cigarette smoke condensate and heavy metals that are present in tobacco smoke may reduce genomic DNA methylation [54]. Although the exact mechanism by which tobacco carcinogens affects global DNA methylation is still unknown, the buccal mucosal cells of tobacco smokers were shown to have a reduced concentration of folate [55], which is required for the maintenance of methylation patterns in DNA [56]. In addition, it has been reported that chemical components present in tobacco smoke can decrease folic acid and vitamin B_12_ levels [57], thus suggesting they may alter the activity of the enzymatic pathways required for DNA methylation. In our study, OPSCC current smokers who relapsed within 2 years exhibited reduced LINE-1 methylation levels when compared to former and never smokers. Interestingly, the association between smoking habits and LINE-1 hypomethylation was stronger in HPV16-negative OPSCC cases, where tobacco exposure and alcohol consumption represent the major etiologic risk factor. Our observation suggests that distinct molecular phenotypes may characterize relapse in HPV16-negative OPSCC compared to those arising from HPV16 infection. Thus, within the poor prognosis of HPV16-negative tumors, low LINE-1 methylation could identify a subset of particularly high-risk patients that could benefit from enhanced post-treatment surveillance. These findings may highlight hypomethylation of LINE-1 as a promising biomarker for prospective studies specifically focused on HPV-negative OPSCC patients. In fact, the management of patients with resectable, locally advanced high-risk OPSCC is complex, and new predictive tools are awaited for selecting between surgical and nonsurgical approaches in this setting.

In the last years, LINE-1 methylation level has been widely considered as a surrogate marker for global DNA methylation [36], and genomic DNA hypomethylation, indicated by LINE-1 hypomethylation, has been frequently associated with chromosomal instability which represents a characteristic phenotype of more invasive cancers having worse prognosis [58]. However, some studies suggest LINE-1 hypomethylation may have itself some biologic effects in promoting tumor progression. This would occur through transcription of specific genomic portions within the promoter of hypomethylated LINE-1 elements that can lead to the aberrant expression of proto-oncogenes and/or non-coding RNA able to enhance the tumor metastatic potential [59, 60]. Although our findings may suggest a role for LINE-1 elements in tumor progression, further studies are required to specifically investigate the potential mechanisms by which genome-wide DNA hypomethylation may affect the clinical course of OPSCC, especially in HPV-negative patients.

Despite our results emphasize the prognostic role of LINE-1 hypomethylation in early OPSCC relapse, our study has some limitations. First of all, some factors that may have a significant impact on genome-wide DNA methylation (i.e., alcohol consumption, diet, and physical activity) [61, 62] were not evaluated since the study population was not prospectively recruited, and some patient-related information could not be recorded at surgery. Further, for smokers, we did not have information on the amount of cigarettes smoked per day, so it was not possible to calculate their “pack year.” Therefore, current smoking was considered for evaluation of OPSCC relapse risk profile. Second, the number of OPSCC patients enrolled in this study was limited, precluding sufficient power in subgroup analyses. Therefore, future prospective studies are warranted to validate the prognostic potential of LINE-1 methylation in a larger cohort of OPSCC patients. Furthermore, the accurate methylation analysis of LINE-1 repetitive DNA elements in patients’ tissues before and after OPSCC relapse would improve our understanding of its mechanistic role in tumor biology and further support its prognostic significance.

## Conclusions

In summary, our results highlight the role of LINE-1 hypomethylation in identifying OPSCC patients at higher risk for early relapse, independently of other classic risk factors. Accordingly, our findings suggest that the evaluation of LINE-1 methylation status may greatly help in guiding the post-treatment surveillance and/or the choice of treatment intensity in OPSCC patients belonging to the hypomethylated group. Future prospective studies with larger sample populations need to be performed to confirm our findings.

## Abbreviations

CH: Surgery
CI: Confidence intervals
CT: Chemotherapy
DNMTs: DNA methyltransferases
HNSCC: Head and neck squamous cell carcinoma
HR α-HPV: High-risk alpha human papillomavirus
LINE-1: Long interspersed nucleotide element-1
N: Number of lymph nodes involved
OPSCC: Oropharyngeal squamous cell carcinoma
OR: Odds ratios
QMSP: Quantitative methylation-specific PCR
RT: Radiotherapy
T: Tumor size
